# The impact of health literacy on diagnosis and outcomes of *symptomatic* cancer *by ethnicity*: a systematic review protocol

**DOI:** 10.1186/s13643-018-0831-5

**Published:** 2018-10-17

**Authors:** Bogdan Chiva Giurca, William Hamilton, Tanimola Martins

**Affiliations:** University of Exeter Medical School, Room G02 Medical School Building, St Luke’s Campus, Heavitree Road, Exeter, EX1 2LU UK

**Keywords:** Health inequalities, Health literacy, Ethnicity, Cancer diagnosis, Cancer outcomes

## Abstract

**Background:**

Ethnic minorities in multi-ethnic societies like the UK and USA have poorer outcomes for some cancer types when compared with the majority. The causes of ethnic inequalities in cancer outcomes are complex and not fully understood. In particular, the potential role of health literacy on symptomatic presentation and diagnostic interval (the period between first consultation within primary care and definitive diagnosis of cancer) by ethnicity is unknown. Given the increasing need for shared decision-making and patient involvement in the diagnostic process, understanding the potential impact of the differences in health literacy may help redress ethnic inequality in cancer outcomes. The present study aims to critically examine the evidence in this area.

**Methods:**

Seven electronic databases will be searched using keywords and controlled vocabulary related to ethnicity, health literacy, cancer diagnosis and cancer outcomes. Citations and bibliography searches of included studies will be performed to identify relevant studies that have cited eligible articles. Authors of included studies will be contacted to identify unpublished studies. Eligible studies will be restricted to primary cancers. Study quality will be evaluated in using the Critical Appraisal Skills Programme (CASP) checklists. A descriptive summary of selected studies will be presented, and the synthesis will follow a narrative framework.

**Discussion:**

This systematic review will summarise the evidence regarding ethnic inequality in health literacy and how this impacts on diagnosis and outcomes of cancer. The review will identify possible areas for future research, and inform clinical practice and interventions to reduce ethnic inequalities in cancer diagnosis and outcomes.

**Electronic supplementary material:**

The online version of this article (10.1186/s13643-018-0831-5) contains supplementary material, which is available to authorized users.

## Background

Ethnic minorities in multi-ethnic societies such as the UK and USA have poorer outcomes for some cancer types when compared with the majority [1–4]. Evidence from the USA (where historical data on ethnicity exists) shows that non-Hispanic Black Americans have the highest mortality for nearly all major cancer types compared to other ethnic groups [5]. In the UK, despite major limitations to data on ethnicity, a recent report shows that Asian-Pakistani, Black African and Caribbean people have a higher proportion of advanced-stage lung cancer compared to their British White counterparts, with the black groups also having higher proportions of advanced-stage female breast and colorectal cancers [6]. Advanced-stage at diagnosis is strongly associated with lower cancer survival and is thought to contribute significantly to the UK’s poorer cancer outcomes relative to other developed countries [7, 8]. Efforts to improve cancer outcomes in the UK are largely geared towards promoting early diagnosis of cancer; minimising ethnic inequalities is considered a key aspect to this [9].

However, the causes of ethnic inequalities in cancer diagnosis are complex and not fully understood [1]. Evidence shows that when compared to the majority, ethnic minorities have poorer awareness of symptoms and are less likely to accept screening. Additionally, once symptoms have occurred, a systematic review showed that these minority groups are more likely to delay primary care consultation and may experience unduly prolonged diagnostic interval in female breast, oesophageal, lung and colorectal cancers [10]. We have also shown in a vignette-based study that Black men were less willing to accept prostate-specific antigen (PSA) testing and digital rectal examination (DRE) when presented with hypothetical scenarios about prostate cancer risks, symptoms, investigations and possible prognoses [11]. Some of these inequalities may be related to differences across ethnic groups in health literacy, although few UK studies have specifically examined this subject area.

Health literacy refers to a set of cognitive and social skills required to understand, access and use information in ways which promote and maintain good health [12]. In England, recent data shows that an estimated 43% of adults aged 16–65 years have limited literacy in health [13]. Indeed, when both literacy and the numeracy components of health materials were combined, around 60% of all English adults were unable to understand oral and written health information received from their doctor or awareness campaign materials [13]. Limited health literacy has been linked to poorer health status, advanced-stage at diagnosis, higher mortality rates and decreased involvement in clinical trials [14–16]. In relation to ethnicity, a number of studies, particularly in the UK and the USA have demonstrated that ethnic minority groups are at a higher risk of being below the average health literacy threshold [13, 17, 18].

However, the extent to which this may contribute to ethnic inequalities in diagnosis and outcomes of symptomatic cancer is uncertain. Given the increasing need for shared decision-making and patient involvement in the diagnostic process, understanding the potential impact of ethnic differences in health literacy is important. The present study aims to critically examine the evidence in this area.

## Methods

### Design

This systematic review will be guided by AMSTAR, [19] and the reporting will follow the Preferred Reporting Items for Systematic Reviews and Meta-Analysis (PRISMA) framework (see Additional file 1) [20].

#### Eligibility criteria

##### Type of studies

We will include studies that examined ethnic differences in health literacy and how this impacts primary care consultation, diagnostic interval and outcomes of symptomatic cancer. For simplicity, diagnostic interval will be separated into three time periods using the Aarhus statement for cancer diagnostic studies: [21] (a) primary care interval (period between first symptomatic presentation and primary care investigation); (b) referral interval (period between primary care investigation and specialist referral); and (c) secondary care interval (period between first specialist presentation and diagnosis) [21]. We anticipate that our final studies will vary in terms of design, with the majority being observational studies. However, studies will not be excluded based on design, and all types of studies (quantitative, qualitative and mixed-methods) will be eligible for inclusion.

##### Study population

The target population for this review includes everyone with a diagnosis of cancer. Eligible studies will be restricted to primary cancers; studies focusing on metastatic cancers from previous cancers will be excluded. The effect of health literacy on primary care consultation and diagnostic interval are likely to be more significant in primary cancers than secondary cancers. Studies will not be excluded based on the cancer sites studied, but only studies published in English language will be eligible.

##### Exposure ethnic groups

The exposure ethnic groups will comprise the minority ethnic groups in the UK and in countries with similar health care system in terms of cost, availability and access (e.g., Denmark, New Zealand, Australia and Canada). In most societies, the minority ethnic group forms less than half of the population and enjoys only a limited access to roles central to the economy and political system of the society [22]. In this review, we will define ethnicity in line with the UK’s national census definition, which to a large extent includes all major ethnic groupings around the world (see Additional file 2).

Furthermore, in our literature search, we will include broader terms such as race, ethnicity, ethnic groups, ethnic majority and ethnic minority to capture ethnic groups not listed in the UK’s national census definition. We will exclude studies conducted in South America, Africa, Asia and the Middle East due to the fundamental differences in the organisation and delivery of health care in these countries compared to the UK and other developed countries. Access to healthcare in many low-income countries is based on out-of-pocket recompense, whereas most developed countries tend to have a mixture of publicly funded and some form of insurance scheme at the very least [23, 24]. Also, the distinction between primary care and secondary care is often obscure in developing countries compared to the developed countries. Therefore, synthesising results of studies conducted in these very different healthcare systems may prove difficult if not inappropriate. Selected studies will include those conducted in countries within the International Cancer Benchmarking Partnership, which seeks to explain international differences in cancer outcomes [7].

##### Comparison ethnic group

The comparison ethnic group will be the majority ethnic groups in the UK and in countries with similar healthcare system as stated above. In most societies, the majority ethnic groups constitutes over half of the population and owns the power to function as reward allocators in addition to being the custodians and sustainers of the dominant value system [22]. Similar to our definition of the exposure ethnic groups, we will use the definition in the UK’s national census.

##### Outcome measures

The primary outcomes of interest will include (a) ethnic differences in health literacy levels (high/low or adequate/inadequate), and (b) how these impact on promptness to primary care consultation and specific intervals of cancer diagnosis. The secondary outcomes will include ethnic differences in health literacy and cancer mortality and survival. The definition of health literacy is complex and fluid. Research in this area has focused largely on ‘functional health literacy’, which reflects basic reading and writing skills [25, 26]. Two other dimensions of health literacy have received increasing attention in recent decades: interactive and critical health literacy. Interactive health literacy refers to the skills required to extract and derive meaning from various health information sources, and the ability to apply the same in real-life situations [25, 26]. Critical health literacy, on the other hand, relates to the cognitive and social skills required to critically evaluate and determine the applicability of health information to personal situations [25, 26]. While all three dimensions are equally important, not all are quantifiable. This study will focus on functional and interactive health literacy, although we will not exclude any study based on health literacy measure.

#### Search strategy

The following online databases will be searched: Medical literature analysis and retrieval system online (MEDLINE), Excerpta Medica DataBase (EMBASE), Web of Science, Psychology Information Database (PsychINFO), Elsevier Bibliographic Database (Scopus), ProQuest Applied Social Sciences Index and Abstracts (ASSIA) and the Cumulative Index to Nursing and Allied Health Literature (CINAHL). These databases will be searched for relevant peer-reviewed articles published from 2000 onwards. In the years since then, ethnic inequality in many other spheres of interest has been increasingly recognised. This recognition has been followed by better recording and reporting of ethnicity in research outputs. The search terms will include controlled vocabulary and keywords relating to the target population, exposure ethnic groups, comparison ethnic group, and outcome measures (see Additional file 2). Citations and bibliography searches of included studies will be performed to identify relevant studies that have cited eligible articles. Relevant grey literatures will be identified from ProQuest Dissertations and Theses Global and OCLC PapersFirst. Online database searches will be re-run once the final review is completed to identify papers emerging after the initial literature search.

#### Study selection

This will involve a two-stage screening process. Firstly, the eligibility criteria will be applied by two independent reviewers (BCG and TM) to screen all titles and abstracts to identify potentially relevant studies. Studies that do not meet the eligibility criteria will be rejected at this stage. Secondly, full-text copies of the remaining articles will be reviewed by the same reviewers independently to identify final selection. Disagreement between the reviewers will be resolved by consensus. If this cannot be achieved, WH will give a third view. We will detail this selection process in a flow-diagram using the PRISMA framework.

#### Quality assessment

Two reviewers (TM and WH) will assess the methodological quality of eligible studies using the Critical Appraisal Skills Programme (CASP) checklists. The CASP is simple, widely used and is available for various study designs including randomised controlled trials, cohort studies, case-control studies, qualitative studies and systematic reviews [27]. Each checklist contains multiple choice questions relating to the validity of studies, significance of the study results and their application to the research needs. TM and WH will select the appropriate CASP checklist based on study designs. Both reviewers will independently appraise and rate each eligible study as “satisfactory”, “medium” or “high-quality”, depending on the extent to which the checklist items are met. Discrepancies will be resolved by discussion with the whole reviewing team, but studies will not be excluded based on quality.

#### Data extraction

Two reviewers (BCG and TM) will independently extract data from all included studies. Extracted data will be added into a data extraction spreadsheet. Five studies will be used to pilot the spreadsheet which will be modified if required before full data extraction begins. Differences between extracted variables will be resolved by consensus. Attempts will be made to acquire missing information by contacting authors of included studies. Data extraction will include study characteristics such as ethnicity, study design, participants’ characteristics, cancer type, health literacy definition and measures, primary care consultation, diagnostic intervals, mortality and survival.

#### Data synthesis

Specific characteristics and findings of the reviewed studies will be illustrated in tables and figures. We anticipate that studies exploring ethnicity and health literacy will be fundamentally different on various grounds such as participants, ethnic affiliation and measures of health literacy. Therefore, a narrative synthesis will be adopted, using the framework of Rodgers and colleagues [28].

#### Dissemination plans

Publication in a relevant peer-reviewed journal and dissemination at national and international conferences. This review has not been registered with PROSPERO.

## Discussion

Empirical evidence is limited in relation to ethnic inequality in diagnosis and outcomes of symptomatic cancer, particularly in the context of a universal health care system such as the UK’s National Health Service. This systematic review will identify and critically evaluate the evidence regarding the impact of health literacy on primary care consultation, diagnostic intervals and outcomes of symptomatic cancer across ethnic groups. To the best of our knowledge, this is the first review in the UK to examine this subject. The involvement of two independent reviewers in screening, data extraction and quality appraisal will enhance the reliability of the conclusions drawn.

Conversely, the exclusion of studies conducted in South America, Africa, Asia and the Middle East due to the differences in healthcare systems will limit the findings of this review. It may be very difficult and inappropriate to synthesise results of studies conducted in these very different healthcare systems. This review may also be limited by publication bias as studies showing no association between health literacy and cancer diagnostic delays by ethnic groups may fail to be published, potentially leaving us with disproportionately positive studies.

Overall, we anticipate that as well as contributing to knowledge, the findings of this review may help shape future interventions to reduce ethnic inequalities in cancer diagnosis and outcomes.

## Additional files

Additional file 1: Completed PRISMA-P Checklist. (DOCX 33 kb)

Additional file 2: **Table S1.** Search terms and Keywords. (DOCX 16 kb)

## Abbreviations

AMSTAR: A Measurement Tool to Assess Systematic Reviews
ASSIA: Applied Social Sciences Index and Abstracts
CASP: Critical appraisal skills programme
CINAHL: Cumulative index to nursing and allied health literature
EMBASE: Excerpta Medica DataBase
MEDLINE: Medical literature analysis and retrieval system online
OCLC: Online Computer Library Center
PRISMA: Preferred reporting items for systematic reviews and meta-analysis
PsychINFO: Psychology Information Database
