# Supplementary material for: The impact of health literacy on diagnosis and outcomes of symptomatic cancer by ethnicity: a systematic review protocol
Source: Syst Rev. 2018 Oct 17;7:164. doi: 10.1186/s13643-018-0831-5 (PMC6192147; doi:10.1186/s13643-018-0831-5)
Supplement: Supplementary file 2 — Table S1. Search terms and Keywords. (DOCX 16 kb) [file 13643_2018_831_MOESM2_ESM.docx]

**Table S1: Search terms and Keywords**

| **Population** | **Exposure** | **Comparison** | **Outcome** |
| --- | --- | --- | --- |
| **Terms relating to cancer:** Cancer, Neoplasm, Malignant Neoplasm, tumour, Malignant tumour, Astrocytoma, Adenocarcinoma, Glioma, Mesothelioma, Medulloblastoma, Myeloma, Melanoma, Neuroblastoma, Sarcoma, Nonmelanoma, Osteosarcoma, Teratoma, Seminoma, Hodgkin, Leukaemia, Lymphoma, Retinoblastoma | **Terms defining ethnic minority groups:** Ethnic* Ethnicity, Ethnic groups, Race* Cultural groups, Ethnic minority, White Irish, Gipsy or Irish Traveller, other Whites, Black African, Black*, Black Caribbean, Asian*, Indian, Pakistani, Bangladeshi, Chinese, Mixed race, White and Black Caribbean, White and Black Africa, White and Asian, Other Mixed, Arab, Other ethnic group. | **Ethnic majority:** Ethnic*, Race* Cultural groups, Ethnic majority, White British, Caucasian | **Primary Outcomes**   1. **Terms relating to Health Literacy:**  health literacy, numeracy, literacy, education, health knowledge, health information 2. **Terms relating to Cancer Diagnosis:** Duration of symptom; Interval of symptoms; Time; Delay; Late; Postpone; and Wait to Symptom, Presentation, Attendance, Consultation, Help seeking, Appointment, Diagnos*, Detection, Treatment, Intervention, Referral. Other terms; Gate keeping, primary care cancer diagnosis, late stage, advance*, present*, grade   **Secondary Outcomes:**  Terms relating to Cancer Outcomes: mortality rate, survival rate |
